# The diagnosis of a metastatic breast tumor from ovarian cancer by the succession of a p53 mutation: a case report

**DOI:** 10.1186/s12957-017-1185-5

**Published:** 2017-06-29

**Authors:** Ryutaro Mori, Manabu Futamura, Kasumi Morimitsu, Chiemi Saigo, Tatsuhiko Miyazaki, Kazuhiro Yoshida

**Affiliations:** 1 Department of Surgical Oncology, Graduate School of Medicine, Gifu University, 1-1 Yanagido, Gifu, 501-1194 Japan; 2 Department of Pathology and Translational Research, Graduate School of Medicine, Gifu University, 1-1 Yanagido, Gifu, 501-1194 Japan; 3 Pathology Division, Gifu University Hospital, 1-1 Yanagido, Gifu, 501-1194 Japan

**Keywords:** Secondary breast neoplasm, Ovarian neoplasm, p53 mutation

## Abstract

**Background:**

Metastatic breast tumors from other organs are very rare. We herein describe the case of a patient with a metastatic breast tumor due to ovarian cancer who was diagnosed by the succession of a p53 mutation.

**Case presentation:**

The patient was a 59-year-old woman with sigmoid colon stenosis. Diagnostic imaging revealed a pelvic mass, multiple liver tumors, ascites, and multiple swollen para-aortic lymph nodes, suggesting an advanced ovarian tumor. Transverse loop colostomy and partial resection of the greater omentum was performed followed by six cycles of paclitaxel with carboplatin chemotherapy (TC therapy). Her cancer almost disappeared, with the exception of a small tumor in her pelvis. Simple hysterectomy with bilateral salpingo-oophorectomy was performed. Two years and 5 months after the second surgery, a mass was detected in her right breast and simple mastectomy was performed. A histological examination of the tumors from the first surgery revealed infiltrating papillary adenocarcinoma and the solid nest proliferation of atypical cells with comedo necrosis and psammoma bodies. The findings of an immunohistochemical analysis were as follows: cancer antigen 125 (CA125 (+)), cytokeratin 7 (CK7 (+)), cytokeratin 20 (CK20 (−)), p53 (+) and CDX2 (−), estrogen receptor (ER (slightly +)), progesterone receptor (PR (slightly +)), and human epidermal growth factor receptor 2 (HER2 (1+)). The breast tumors presented similar morphological features (ER (−), PR (−), HER2 (−), CA125 (+), CK7 (+), CK20 (−), p53 (+), mammaglobin (−), and GCDFP15 (−)), which were not characteristic of breast cancer. A direct sequencing analysis of p53 revealed a p.V173M mutation in exon 5 in both the breast tumor and the ovarian cancer. It was not detected in normal tissue, suggesting that the breast tumors were metastatic serous adenocarcinomas from ovarian cancer.

**Conclusions:**

A direct sequencing mutation analysis of p53 was useful for distinguishing the primary tumor from the metastatic tumor. We should resect metastatic breast tumors to the extent that is possible because the prognosis of such patients is relatively good.

## Background

The survival of patients with metastatic cancer has improved due to advances in its management [1]. Because patients live longer, unusual metastases such as pancreatic metastases, renal metastases, and breast metastases have been identified [2].

Advanced ovarian cancer typically develops into peritoneal dissemination or lymph node metastasis in the abdominal cavity; distant metastases are rare. However, ovarian cancer occasionally develops into distant metastases to the liver, pleura, or lung, and the prognosis of ovarian cancer patients with distant metastasis is poor [3].

The majority of breast tumors originate from the mammary gland, and most metastatic breast tumors originate from the contralateral breast [4]. Metastatic breast tumors from other organs are very rare.

We herein describe the case of a patient with a metastatic breast tumor due to ovarian cancer who was diagnosed by the succession of a p53 mutation.

## Case presentation

**Fig. 1:**
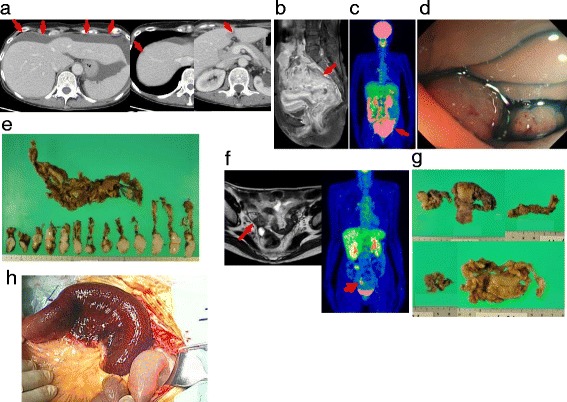
Images and the surgical specimens obtained in the first and second surgeries. **a** CT, **b** MRI, and **c** PET/CT revealed a pelvic tumor involving the sigmoid colon, multiple liver tumors, ascites, and swollen para-aortic lymph nodes. **d** Colonoscopy showed wall edema and stenosis. **e** A specimen obtained by partial resection of the greater omentum. **f** MRI and PET/CT after TC therapy revealed a small pelvic tumor of 2.6 cm in diameter. **g** Specimens obtained by simple hysterectomy with bilateral salpingo-oophorectomy and partial resection of the omentum. **h** The findings at the third surgery for an incidental intestinal obstruction

The patient was a 59-year-old female with a history of cholecystectomy due to gallstones, excision of fibroadenoma in the right breast, and a duodenal ulcer. She suffered from stenosis of the sigmoid colon, and a local doctor referred her to the gynecology and gastroenterology division of our hospital. Computed tomography (CT) revealed a lobulated tumor in her pelvis, multiple tumors in her liver (S4, S6), ascites around her liver, and multiple swollen para-aortic lymph nodes (Fig. 1a). Magnetic resonance imaging (MRI) showed a large pelvic tumor with a cystic component involving the uterus, rectum, and sigmoid colon (Fig. 1b). Positron emission tomography/computed tomography (PET/CT) showed a large tumor in her pelvis and multiple swollen lymph nodes in her abdominal cavity (Fig. 1c). Colonoscopy revealed wall edema and stenosis approximately 30 cm from the anal verge (Fig. 1d). According to these findings, she was diagnosed with advanced ovarian cancer, and transverse loop colostomy and partial resection of the greater omentum were performed (Fig. 1e).

After the first surgery, she received six cycles of paclitaxel with carboplatin chemotherapy (TC therapy), and her cancer almost disappeared, with the exception of a small pelvic tumor of 2.6 cm in diameter (Fig. 1f). Simple hysterectomy with bilateral salpingo-oophorectomy and partial resection of the omentum were performed as a second surgery to remove the residual tumors (Fig. 1g). The pathological diagnosis of the residual tumors in her bilateral ovaries was serous adenocarcinoma.

After the second surgery, the patient received one cycle of TC therapy. Incidentally, she developed an intestinal obstruction 2 months after the second surgery, and partial resection of the small bowel was performed. However, no residual tumor was detected in her abdominal cavity or the resected specimen (Fig. 1h).

**Fig. 2:**
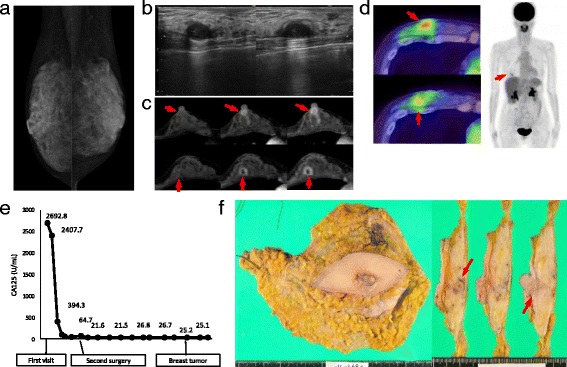
Images of the breast mass, the serum CA125 level, and a specimen obtained by simple mastectomy. No mass was observed on **a** mammography. However, masses under the nipple and near the retromammary space could be detected by **b** US, **c** MRI, and **d** PET/CT. **e** The serum CA125 level was under the upper limit. **f** A specimen obtained by simple mastectomy

Two years and 5 months after the second surgery, the patient visited our division with a mass in her right breast. Mammography did not reveal a tumor (Fig. 2a); however, ultrasonography (US) showed a circumscribed mass near the retromammary space in her right mammary gland (Fig. 2b). MRI (Fig. 2c) and PET/CT (Fig. 2d) showed two masses under the nipple and near the retromammary space in her right breast. The findings of histological examination of a core needle biopsy specimen were consistent with metastasis of serous adenocarcinoma. Because no other metastatic mass was detected on PET/CT and the patient’s serum cancer antigen 125 (CA125) level was below the upper limit (Fig. 2e), simple mastectomy of the right breast was performed (Fig. 2f).

**Fig. 3:**
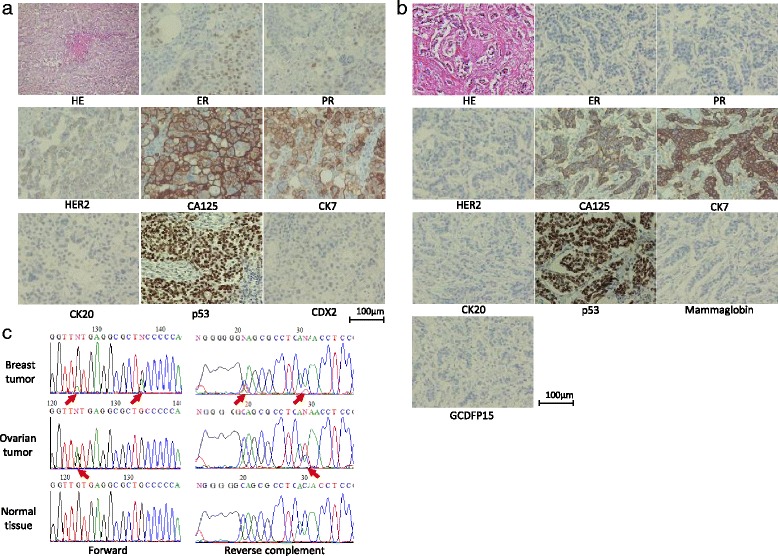
The histopathological and immunohistochemical diagnoses of the omental tumor and the direct sequencing mutation analysis of p53. **a** The omental tumor findings (HE, ER, PR, HER2, CA125, CK7, CK20, p53, and CDX2). **b** The breast tumor findings (HE, ER, PR, HER2, CA125, CK7, CK20, p53, mammaglobin, and GCDFP15). **c** A sequence analysis of exon 5 from the breast tumor, the ovarian tumor, and normal breast tissue. The *arrows* indicate point mutations

According to the findings of histological examinations, the tumors in the greater omentum that were resected in the first surgery demonstrated infiltrating papillary adenocarcinoma and the solid nest proliferation of atypical cells with comedo necrosis and psammoma bodies. The findings of an immunohistochemical analysis were as follows: CA125 (+), cytokeratin 7 (CK7 (+)), cytokeratin 20 (CK20 (−)), p53 (+), and caudal-type homeobox protein 2 (CDX2 (−)). This was consistent with metastatic serous adenocarcinoma from ovarian cancer. After mastectomy, the findings of an additional immunohistochemical analysis of the tumor were as follows: estrogen receptor (ER (slightly +)), progesterone receptor (PR (slightly +)), and human epidermal growth factor receptor 2 (HER2 (1+)) (Fig. 3a). Moreover, the breast tumors also presented solid nest formation and comedo necrosis with stromal invasion and the following findings: ER (−), PR (−), HER2 (−), CA125 (+), CK7 (+), CK20 (−), p53 (+), mammaglobin (−), and gross cystic disease fluid protein 15 (GCDFP15 (−)) (Fig. 3b). These findings are not characteristic of breast cancer but were similar to the tumor of the omentum. We also performed a direct sequencing mutation analysis of p53 to confirm the succession of the mutation. DNA was extracted from formalin-fixed, paraffin-embedded samples of the breast tumor, the ovarian tumor, and normal breast tissue using a NucleoSpin DNA FFPE XS system (Takara Bio Inc., Japan), and exons 5–8 of the p53 were amplified by a polymerase chain reaction (98, 56, and 72 °C for 10, 30, and 60 s, respectively) in a Premix Ex Taq® Hot Start Version system (Takara Bio Inc.) using specific primers (Table 1) that were designed based on the data of previous studies [5]. The products were sent to the Genomics Research division of Gifu University, and a direct sequencing analysis was performed. The results showed that both the breast tumor and the ovarian cancer had a p.V173M mutation in exon 5, which was not detected in normal tissue. The breast tumor also had an additional p.C176Y mutation (Fig. 3c). According to these findings, the breast tumors were confirmed to be metastatic serous adenocarcinoma from ovarian cancer.

**Table 1:** The primer sequences for the polymerase chain reaction

Exon	Primer sequence
5	5′-TTCCTCTTCCTGCAGTACTCC-3′
	5′-GCCCCAGCTGCTCACCATCG-3′
6	5′-CACTGATTGCTCTTAGGTCT-3′
	5′-AGTTGCAAACCAGACCTCAGG-3′
7	5′-TCTCCTAGGTTGGCTCTGAC-3′
	5′-CAAGTGGCTCCTGACCTGGA-3′
8	5′-CCTATCCTGAGTAGTGGTAA-3′
	5′-CCTGCTTGCTTACCTCG-3′

## Discussion

We encountered a patient with a metastatic breast tumor from ovarian cancer. This diagnosis was made according to the morphological features, the results of an immunohistochemical analysis, and the succession of a p53 mutation.

Metastasis from other organs accounts for only 0.43% of all cases of malignant breast tumors [4]. However, Di Bonito et al. previously reported 12 cases with metastatic breast tumors that were detected on autopsy. Only two cases were diagnosed before death [6]. Thus, many cases of metastatic breast tumor may be missed before death. DeLair et al. reported 85 cases of metastatic breast tumors, the origins of which were sarcoma (*n* = 18), melanoma (*n* = 18), ovarian cancer (*n* = 14), lung cancer (*n* = 11), and gastrointestinal cancer (*n* = 17) [7]. Carcinoma most commonly originates in the ovaries. We performed a PubMed database search for case reports on metastatic breast tumors and identified 37 case reports. The majority (25 reports) described unilateral breast tumors. However, bilateral (four reports) [8], inflammatory (six reports) [9], and ductal carcinoma in situ-like (one report) [10] metastatic breast tumors were also described. Karam et al. reported 10 cases of metastatic breast tumor and noted that the mean interval between the diagnosis of ovarian cancer and breast/axilla events was 70.7 months and that the median overall survival after a breast event was 26 months, suggesting that metastatic breast tumors from ovarian cancer are not associated with a poor prognosis [11].

The differential diagnosis between primary breast cancer and a metastatic breast tumor from ovarian cancer is not simple. Mammaglobin and gross cystic disease fluid protein 15 (GCDFP15) are known to be breast cancer-specific markers. Bhargava et al. reported that the rates of mammaglobin and GCDFP positivity (including any strength of stainability) were 93.1% (54/58) and 84.5% (49/58), respectively, and that only two cases were negative for both markers. Furthermore, among 40 cases of ovarian serous carcinoma, only one case showed mammaglobin positivity (patchy, moderate staining) [12]. CA125 is a well-known tumor marker of ovarian cancer, and rates of CA125 positivity detected in ovarian cancer and primary breast cancer specimens by immunohistochemistry were 90% (38/42) (strong and diffuse staining) and 16% (6/36) (focal and weak staining), respectively [13]. The expression pattern of mammaglobin, GCDFP15, and CA125 are summarized in Table 2. In our case, the breast tumor was negative for mammaglobin and GCDFP15, while both the breast and the ovarian cancers were positive for CA125, suggesting that the breast tumor had metastasized from ovarian cancer.

**Table 2:** The expression pattern of mammaglobin, GCDFP15, and CA125

	Breast cancer (%)	Ovarian cancer
Negative for both mammaglobin and GCDFP15	3	
Mammaglobin-positive	93.1	0.25
GCDFP15-positive	84.5	
CA125-positive	16	90

The p53 gene has various mutation points in exons 5–8, and the succession of the p53 mutation pattern can be used to distinguish primary tumors from metastatic tumors [14–16]. In our case, the p.V173M in the ovarian cancer was observed to have succeeded to the breast tumor, strongly suggesting that the breast tumor had metastasized from ovarian cancer.

## Conclusion

We encountered the case of a patient with a breast tumor that had metastasized from ovarian cancer. A direct sequencing mutation analysis of p53 was useful for distinguishing the primary tumor from the metastatic tumor. We should resect metastatic breast tumors to the extent that is possible because the prognosis of such patients is relatively good.

## Abbreviations

CA125: Cancer antigen 125
CDX: Caudal-type homeobox protein 2
CK20: Cytokeratin 20
CK7: Cytokeratin 7
CT: Computed tomography
ER: Estrogen receptor
GCDFP15: Gross cystic disease fluid protein 15
HER2: Human epidermal growth factor receptor 2
MRI: Magnetic resonance imaging
PET/CT: Positron emission tomography/computed tomography
PR: Progesterone receptor
